# Design of a MCoTI-Based Cyclotide with Angiotensin (1-7)-Like Activity

**DOI:** 10.3390/molecules21020152

**Published:** 2016-01-26

**Authors:** Teshome Aboye, Christopher J. Meeks, Subhabrata Majumder, Alexander Shekhtman, Kathleen Rodgers, Julio A. Camarero

**Affiliations:** 1 Department of Pharmacology and Pharmaceutical Sciences, University of Southern California, Los Angeles, CA 90089-9121, USA; aboye@usc.edu (T.A.); meeksc@usc.edu (C.J.M.); krodgers@usc.edu (K.R.); 2 Department of Chemistry, State University of New York, Albany, NY 12222, USA; smajumder@albany.edu (S.M.); ashekhtman@albany.edu (A.S.); 3 Department of Chemistry, University of Southern California, Los Angeles, CA 90089-9121, USA

**Keywords:** cyclotide, MAS1 receptor, angiotensin

## Abstract

We report for the first time the design and synthesis of a novel cyclotide able to activate the unique receptor of angiotensin (1-7) (AT1-7), the MAS1 receptor. This was accomplished by grafting an AT1-7 peptide analog onto loop 6 of cyclotide MCoTI-I using isopeptide bonds to preserve the α-amino and *C*-terminal carboxylate groups of AT1-7, which are required for activity. The resulting cyclotide construct was able to adopt a cyclotide-like conformation and showed similar activity to that of AT1-7. This cyclotide also showed high stability in human serum thereby providing a promising lead compound for the design of a novel type of peptide-based in the treatment of cancer and myocardial infarction.

## 1. Introduction

Modulation of the renin-angiotensin system (RAS) by angiotensin (1-7) (AT1-7) is recently emerged as an attractive and novel chemotherapeutic and chemopreventive treatment for lung cancer [1]. AT1-7 is a component of the renin-angiotensin system (RAS) with vasodilator, anti-proliferative and anti-angiogenic properties [1,2,3,4,5]. Recent studies have shown that AT1-7 is able to reduce serum-stimulated growth of human lung cancer cells both *in vitro* and *in vivo* through activation of the unique AT1-7 receptor, MAS1 [3]. In results with human lung adenocarcinoma xenografts, AT1-7 was able to inhibit tumor growth through reduction in cyclooxygenase-2 (COX-2) activity and production of pro-inflammatory prostaglandins [2]. In contrast, AT1-7 had no effect on cyclooxygenase 1 (COX-1) activity in the same xenograft tumor. All these suggest that selective activation of the MAS1 receptor may represent a novel treatment for lung cancer through reduction of COX-2 activity [2]. Unfortunately, AT1-7 has a limited clinical potential due to its unfavorable pharmacokinetic profile.

**Figure 1:**
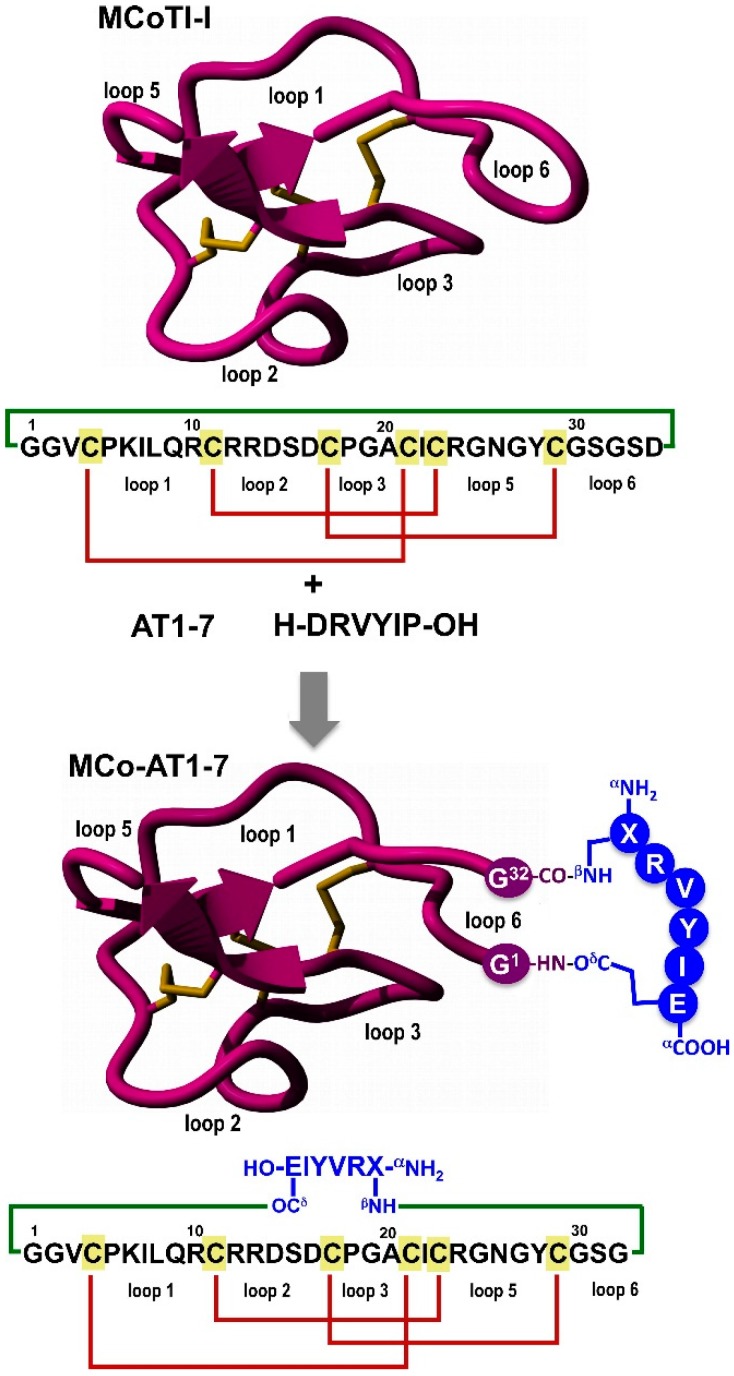
Design of grafted cyclotide MCo-AT1-7 to activate the receptor MAS1. The upper part of the panel shows the primary and tertiary structures of the cyclotide MCoTI-I (structure is based on a homology model using the solution structure of MCoTI-II as template (PDB: 1IB9) [14]), and the primary structure of peptide angiotensin 1-7 (AT1-7). The cyclized polypeptide is stabilized by the three-disulfide bonds (shown in red). The lower part of the figure shows the strategy used to graft an AT1-7-derived peptide onto the loop 6 of cyclotide MCoTI-I. The AT1-7-derived peptide was linked to the cyclotide backbone through the side-side chains of the *N*- and *C*-terminal residues forming two isopeptide bonds. The sequence corresponding to the AT1-7-derived peptides is shown in blue. Residue X represents l-2,3-diaminopropionic acid. Molecular graphics were built with Yasara (www.yasara.org).

Cyclotides are small globular microproteins ranging from 28 to 37 amino acids with a unique head-to-tail cyclized backbone topology that is stabilized by three disulfide bonds (Figure 1) [6,7,8]. The number and positions of cysteine residues are conserved throughout the family, forming the cyclic cystine-knot (CCK) motif [6] that acts as a highly stable and versatile framework on which hyper-variable loops are arranged. This cyclic cystine-knot (CCK) framework provides an extremely rigid molecular scaffold [9,10] with exceptional to resistance to thermal, chemical and biological degradation [7,8]. Cyclotides can be considered as natural combinatorial peptide libraries structurally constrained by the cystine-knot scaffold and head-to-tail cyclization but in which hypermutation of essentially all residues is permitted with the exception of the strictly conserved cysteines that comprise the knot [11,12,13].

Naturally-occurring cyclotides have shown to possess various pharmacologically-relevant activities [7,15], and have been reported to be able to cross mammalian cell membranes [16,17] to antagonize intracellular protein-protein interactions in animal models [18]. The main features of cyclotides are a remarkable stability due to the CCK framework, a small size making them readily accessible to chemical synthesis, and an excellent tolerance to sequence variations. Altogether, these features make the cyclotide scaffold an excellent molecular framework for the design of novel peptide-based therapeutics [8,19] making them ideal substrates for molecular grafting of biological peptide epitopes [18,20,21,22].

The peptide AT1-7 is a hormone that in general counteracts the angiotensin II through its own signaling pathway involving the MAS receptor. Studies in animal models show that AT1-7 has ample therapeutic potential in cardiovascular disease [23,24], and more recently in lung cancer chemotherapy and chemoprevention [2,3]. Despite its potential therapeutic value, AT1-7 does not offer ideal prospects for clinical use due to its poor pharmacodynamics and pharmacokinetic properties, mostly due to its rapid degradation in plasma [25].

We report here for the first time the design and synthesis of an engineered cyclotide with similar biological activity to that of the peptide AT1-7. The engineered cyclotide was able to fold correctly and showed high resistance to degradation by human serum therefore providing a promising new peptide-based lead for the treatment of cancer and myocardial infarction.

## 2. Results and Discussion

In order to produce a novel cyclotide with MAS1 agonistic activity, we used the cyclotide MCoTI-I as molecular scaffold (Figure 1). MCoTI-cyclotides are found in the dormant seeds of the plant *Momordica cochinchinensis*, and are potent trypsin inhibitors (*K_i_* ≈ 25 pM) [26]. Natively-folded MCoTI-cyclotides can be easily produced by standard recombinant methods [27,28,29] as well as by chemical synthesis [18,20], and can also be easily engineered to incorporate novel biological functions [18,20,21,22]. In addition, MCoTI-cyclotides show very little toxicity to human cells (IC_50_ > 100 µM) [18] and therefore represent a desirable molecular scaffold for engineering new compounds with unique biological properties.

To engineer the cyclotide MCoTI-I to have AT1-7 activity, we grafted a derivative of the peptide AT1-7 peptide onto the cyclotide scaffold using loop 6 (Figure 1). This loop has been shown previously to be more disordered in solution [9] and amenable to sequence variation [30].

The peptide was grafted using the side-chains of residues 1 and 7. For this purpose the original residues at Asp1 and Pro7 in the AT1-7 peptide were replaced by diaminopropionic acid and glutamic acid, respectively. These positions have been shown to tolerate mutations in angiotensin-peptides without affecting their biological activity [31,32]. For example Asp1 and Pro7 have been replaced by glutamine and cysteine without negatively affecting the biological activity of the corresponding angiotensin-derived peptides [31,32]. The AT1-7 derived peptide was grafted into the cyclotide backbone between residues Gly1 and Ser32 using the β-amino and γ-carboxylic groups through the creation of two isopeptide bonds (Figure 1). This allowed keeping the native positive and negative charged groups at the *N*- and *C*-terminal of the grafted AT1-7 derivative. The resulting grafted cyclotide was called MCo-AT1-7 (Figure 1).

**Figure 2:**
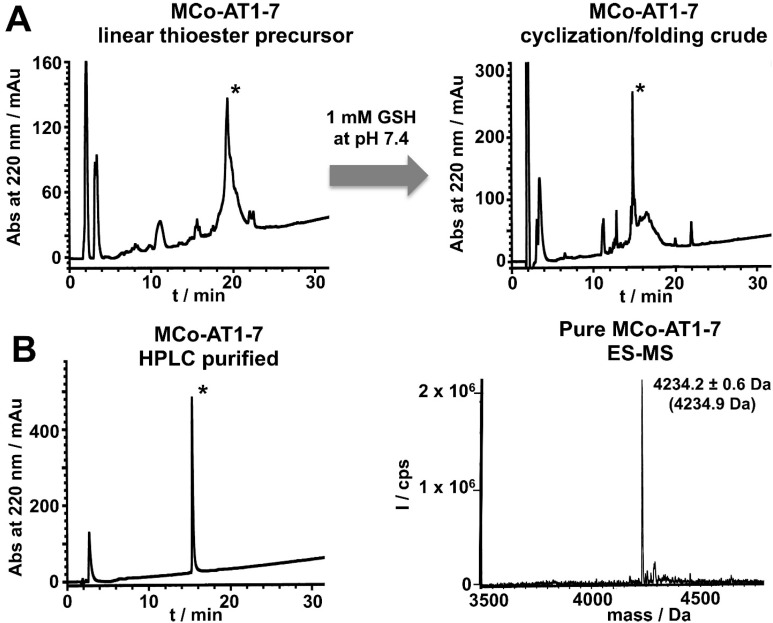
Chemical synthesis and characterization of cyclotide MCo-AT1-7. (**A**) Analytical HPLC traces of for the linear thioester precursor, GSH-induced cyclization/folding crude after 24 h and purified cyclotide. An asterisk indicates the desired peptide; (**B**) Analytical HPLC trace and ES-MS characterization of pure MCo-AT1-7. The expected average molecular weight is shown in parenthesis.

**Figure 3:**
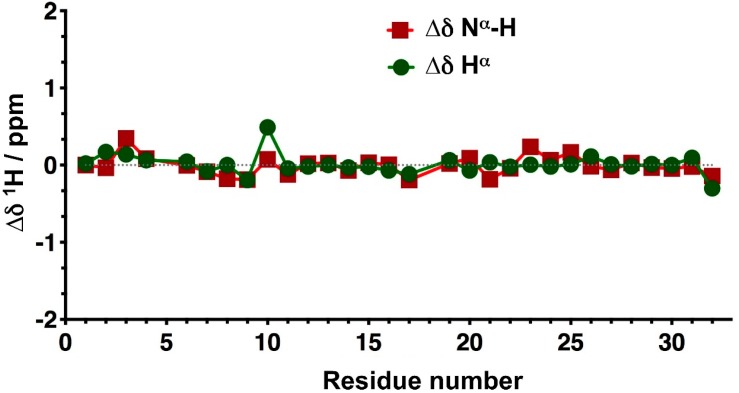
^1^H-NMR characterization of folded cyclotide MCo-AT1-7. Chemical shifts differences of the backbone, Nα-H and Hα protons between the common sequence (residues 1 through 32) of MCoTI-I and MCo-AT1-7 (Table S1).

Cyclotide MCo-AT1-7 was chemically synthesized using Fmoc-based solid-phase peptide synthesis on a sulfonamide resin [16]. Activation of the sulfonamide linker with iodoacetonitrile, followed by cleavage with ethyl mercaptoacetate and acidolytic deprotection, provided the fully deprotected linear peptide α-thioester (Figure 2A). The corresponding peptide thioester precursor was efficiently cyclized and folded in a one-pot reaction using sodium phosphate buffer at pH 7.2 in the presence of 1 mM GSH. The cyclization/folding reaction was complete in 24 h (Figure 2A). The cyclization/folding yields was estimated by HPLC to be ≈35% (Figure 2A). Folded MCo-AT1-7 was purified by reverse-phase HPLC and characterized by ES-MS confirming ≥95% purity according to HPLC (Figure 2B). Cyclotide MCo-AT1-7 was also characterized by ^1^H-NMR indicating that adopts a native cyclotide fold (Figure 3 and Figure S3, and Table S1).

**Figure 4:**
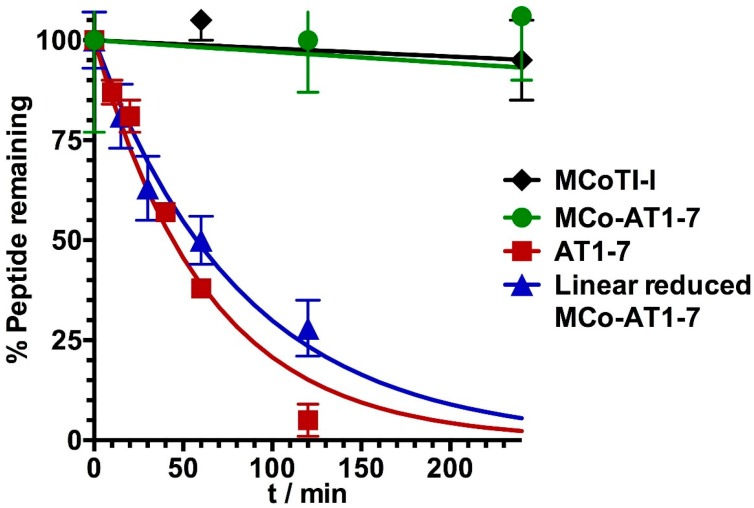
Stability of cyclotides MCo-AT1-7 and MCoTI-I, and peptide AT1-7 and reduced linear MCo-AT1-7 precursor to human serum at 37 °C. Undigested peptides were quantified by HPLC-MS/MS.

We also studied the biological stability of cyclotide MCo-AT1-7 and compare it to that of the empty scaffold (MCoTI-I) and the peptide AT1-7 (Figure 4). This was accomplished by incubating the corresponding peptides in human serum at 37 °C. The quantitative analysis of undigested polypeptides was performed using liquid chromatography coupled with tandem mass spectrometry (LC-MS/MS). Naturally occurring MCoTI-cyclotides present a very rigid structure [9,10], which makes them extremely stable to proteolytic degradation. Remarkably, cyclotide MCo-AT1-7 was only slightly less stable (τ_1/2_ = 39 ± 5 h) than the parent cyclotide MCoTI-I (τ_1/2_ = 55 ± 7 h) (Figure 4 and Figure S2). In contrast, peptide AT1-7 was degraded considerably faster under the same conditions (τ_1/2_ = 44 ± 3 min) (Figure 4 and Figure S2). A linearized, reduced and alkylated version of MCo-AT1-7 (Figure S1) was also rapidly degraded (τ_1/2_ = 57 ± 5 min) (Figure 4 and Figure S2) indicating the importance of the circular Cys-knot topology for proteolytic stability.

**Figure 5:**
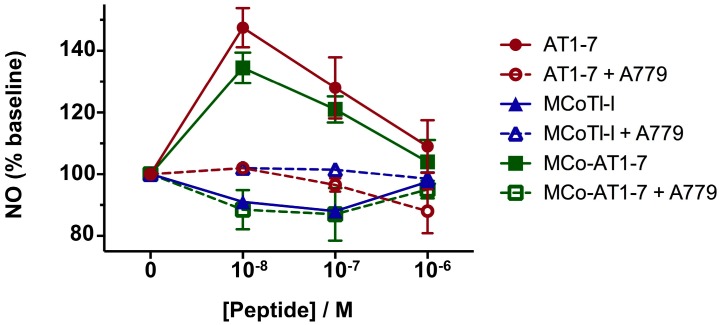
Biological activity of cyclotide MCo-AT1-7. MAS1 stably transfected CHO cells were tested using different concentrations of cyclotide MCo-AT1-7 and peptide AT1-7 in the absence or presence of MAS1 antagonist A779. The amount of intracellular NO was measured by fluorescence as described in the experimental section. Cyclotide MCoTI-I was used as negative control. The average of standard deviation of two experiments is shown.

Next, we tested the ability of cyclotide MCo-AT1-7 to activate the MAS1 receptor using CHO cells stably transfected with pTEJ-8 vector expressing recombinant human MAS1 in a cell-based fluorescence assay to detect the amount of NO release (Figure 5). Cyclotide MCo-AT1-7 and peptide AT1-7 (used as positive control) were able to increase the intracellular concentration of NO in a dose dependent manner as measured by the level of fluorescence. As expected, the naturally-occurring cyclotide MCoTI-I did not show any increase in intracellular NO levels (Figure 5). When the MAS1 activation biological assay was performed in the presence of the MAS1 peptide antagonist A779, no increase in the intracellular concentration of NO was detected therefore confirming the agonistic activity of the cyclotide MCo-AT1-7. A similar profile was obtained with the peptide AT1-7 (Figure 5). The activity of the cyclotide MCo-AT1-7 was estimated to be ≈90% of that of peptide AT1-7 at 10 nM in this assay.

## 3. Discussion

In summary we report here for the first time the design and synthesis of a novel cyclotide able to efficiently activate the MAS1 receptor. This was successfully accomplished by grafting an AT1-7-derived peptide onto loop 6 of the cyclotide MCoTI-I using the side-chains of the first and last residues of the grafted peptide through the formation of isopeptide bonds (Figure 1). ^1^H-NMR studies also revealed that the grafting of the AT1-7-derived peptide using isopeptide bonds onto this loop did not affect the native cyclotide scaffold, indicating the tolerance of this loop for the grafting of peptide sequences using non-native peptide bonds [18,20]. Cyclotide MCo-AT1-7 showed to be a potent MAS1 agonist, with similar activity to that of the peptide AT1-7. It is also worth noting that the cyclotide MCo-AT1-7 showed a remarkable resistance to biological degradation in human serum, with a τ_1/2_ value of ≈39 h. This value is similar to that of the cyclotide MCoTI-I and significantly higher that the half-life of the AT1-7 peptide (τ_1/2_ ≈ 1 h). Although further analysis will be required to evaluate the therapeutic value of these compounds *in vivo*, altogether, our results show that engineered cyclotides hold great promise for the development of a novel type of peptide-based therapeutic able to efficiently target extracellular protein/protein interactions.

## 4. Materials and Methods

### 4.1. General Information

All chemicals involved in synthesis or analysis were obtained from Aldrich (Milwaukee, WI, USA) or Novabiochem (San Diego, CA, USA) unless otherwise indicated. All reactions were performed at room temperature unless indicated otherwise. Analytical HPLC was performed on a HP1100 series instrument (Agilent, Santa Clara, CA, USA) with 220 and 280 nm detection using a Vydac C18 column (5 μm, 4.6 × 150 mm; Grace, Columbia, MD, USA) at a flow rate of 1 mL/min. Preparative and semipreparative HPLC were performed on a Waters Delta Prep system (Waters Co., Milford, MA, USA) fitted with a Waters 2487 UV-visible detector using either a Vydac C18 column (15–20 μm, 50 × 250 mm) or a Vydac C18 (15–20 μm, 10 × 250 mm) at a flow rate of 50 or 5 mL/min, respectively. All runs used linear gradients of 0.1% aqueous trifluoroacetic acid (TFA) (solvent A) *vs.* 0.1% TFA, 90% acetonitrile in H_2_O (solvent B). Ultraviolet-visible (UV-vis) spectroscopy was carried out on an Agilent 8453 diode array spectrophotometer (Agilent). Electrospray mass spectrometry (ES-MS) analysis was routinely applied to all compounds and components of reaction mixtures. ES-MS was performed on an Applied Biosystems API 3000 triple quadrupole electrospray mass spectrometer (Applied Biosystems, Foster City, CA, USA) using Analyst 1.4.2. Calculated masses were obtained using Analyst 1.4.2.

### 4.2. Preparation of Fmoc-Tyr(tBu)-F

For all peptides’ synthesis, Fmoc-Tyr(*t*Bu)-F was prepared using dimethylaminosulfur trifluoride (DAST) and immediately loaded to resin as described previously [16]. Briefly, DAST (160 μL, 1.2 mmol) was added dropwise at 25 °C under nitrogen current to a stirred solution of Fmoc-Tyr(*t*Bu)-OH (459.6 mg, 1 mmol) in dry dichloromethane (DCM, 10 mL), containing dry pyridine (81 µL, 1 mmol). After 20 min, the mixture was washed with ice-cold water (3 × 20 mL). The organic layer was separated and dried over anhydrous MgSO_4_. The solvent was removed under reduced pressure to give the corresponding Fmoc-amino acyl fluoride as yellowish oil that was immediately used.

### 4.3. Loading of 4-Sulfamylbutyryl AM Resin with Fmoc-Tyr(tBu)-F

Loading of the first residue for all peptides was accomplished using Fmoc-Tyr(*t*Bu)-F according to standard protocols [16]. Briefly, 4-sulfamylbutyryl AM resin (420 mg, 0.33 mmol; Novabiochem) was swollen for 30 min with dry DCM and then drained. A solution of Fmoc-Tyr(*t*Bu)-F (≈461 mg, 1 mmol) in dry DCM (2 mL) and di-isopropylethylamine (DIEA, 180 µL, 1 mmol) was added to the drained resin and reacted at 25 °C for 1 h. The resin was washed with dry DCM (5 × 5 mL), dried and kept at –20 °C until use.

### 4.4. Chemical Synthesis of Cyclotide MCo-AT1-7

Solid-phase synthesis of cyclotide MCo-AT1-7 was carried out on an automatic peptide synthesizer ABI433A (Applied Biosystems) using the Fast-Fmoc chemistry with 2-(1*H*-benzotriazol-1-yl)-1,1,3,3-tetramethyluronium hexafluorophosphate (HBTU)/diisopropylethylamine (DIEA) activation protocol at 0.1 mmole scale on a Fmoc-Tyr(*t*Bu)-sulfamylbutyryl AM resin. Side-chain protection compatible with Fmoc-chemistry was employed as previously described for the synthesis of peptide α-thioesters by the Fmoc-protocol [33], except for the *N*-terminal Cys residue, which was introduced as Boc-Cys(Trt)-OH. Following chain assembly, the alkylation, thiolytic cleavage and side chain deprotection were performed as previously described [16]. Briefly, ≈100 mg of protected peptide resin were first alkylated two times with ICH_2_CN (174 µL, 2.4 mmol; previously filtered through basic alumina) and DIEA (82 µL, 0.46 mmol) in *N*-methylpyrrolidone (NMP, 2.2 mL) for 24 h. The resin was then washed with NMP (3 × 5 mL) and DCM (3 × 5 mL). The alkylated peptide resin was cleaved from the resin with HSCH_2_CO_2_Et (200 µL, 1.8 mmol) in the presence of a catalytic amount of sodium thiophenolate (NaSPh, 3 mg, 22 µmol) in dimethylformamide (DMF):DCM (1:2 *v*/*v*, 1.2 mL) for 24 h. The resin was then dried at reduced pressure. The side-chain protecting groups were removed by treating the dried resin with trifluoroacetic acid (TFA):H_2_O:tri-isopropylsilane (TIS) (95:3:2 *v*/*v*, 10 mL) for 3–4 h at room temperature. The resin was filtered and the linear peptide thioester was precipitated in cold Et_2_O. The crude material was dissolved in the minimal amount of H_2_O:MeCN (4:1) containing 0.1% TFA and characterized by HPLC and ES-MS [Expected mass: 4361.1 Da. Observed mass: 4360.0 ± 0.7 Da] as the desired grafted MCoTI-I linear precursor α-thioester (Figure 2). Cyclization and folding was accomplished by flash dilution of the linear α-thioester TFA crude to a final concentration of ≈50 µM into freshly degassed 1 mM reduced glutathione (GSH), 0.1 M sodium phosphate buffer at pH 7.2 for 24 h (Figure 2). The folded cyclotide was purified by semi-preparative HPLC using a linear gradient of 17%–28% solvent B over 30 min. The purified cyclotide was characterized by HPLC and ES-MS confirming ≥95% purity (Figure 2).

### 4.5. Chemical Synthesis of Linearized and S-Alkylated Cyclotide MCo-AT1-7

For the preparation of linearized and S-alkylated MCo-AT1-7, sulfonamide peptide-resin (20 mg) was activated as described above and cleaved from the resin with propylamine in DMF (1:1, 0.8 mL) for 4 h. The resulting peptide was side deprotected and peptide collected by precipitation with diethyl ether as described above. The linear peptide was fully reduced with 1,4-dithioerythritol (40 mM, 300 µL) in freshly degassed 0.1 M Na_2_HPO_4_ buffer at pH 7.5 at 37 °C for 1 h followed by alkylation of all free cysteines with iodoacetamide (0.5 M, 200 µL) for 10 min at 37 °C. The linear and fully reduced cyclotide precursor was purified by semi-preparative HPLC using a linear gradient of 15%–35% solvent B over 30 min. The purified peptide was characterized by HPLC and ES-MS [Expected mass: 4641.2 Da. Observed mass: 4641.1 ± 0.6 Da] (Figure S1).

### 4.6. NMR Spectroscopy

NMR samples were prepared by dissolving cyclotides into 80 mM potassium phosphate pH 6.0 in 90% H_2_O/10% D_2_O (*v*/*v*) to a concentration of approximately 0.5 mM. All ^1^H-NMR data were recorded on an Avance II 700 MHz spectrometer (Bruker, Billerica, MA, USA) equipped with the TXI cryoprobe. Data were acquired at 298 K, and 2,2-dimethyl-2-silapentane-5- sulfonate (DSS), was used as an internal reference. The carrier frequency was centered on the water signal, and the solvent was suppressed by using WATERGATE pulse sequence. ^1^H,^1^H-TOCSY (spin lock time 80 ms) and ^1^H,^1^H-NOESY (mixing time 150 ms) spectra were collected using 4096 t_2_ points and 256 t_1_ of 64 transients. Spectra were processed using Topspin 2.1 (Bruker). Each 2D-data set was apodized by 90°-shifted sinebell-squared in all dimensions, and zero filled to 4096 × 512 points prior to Fourier transformation. Assignments for Hα (H-Cα) and H’ (H-Nα) protons of folded MCo-AT1-7 (Table S1) were obtained using standard procedures [34,35].

### 4.7. Biological Activity of Cyclotide MCo-AT1-7

CHO cells stably transfected with pTEJ-8 vector expressing recombinant human MAS1 clone are grown to confluency. After washing, cells were incubated for a short time in 700 µL of supplemented Tyrode’s salts containing 10 µM 2-phenyl-4,4,5,5-tetramethylimidazoline-1-oxyl-3-oxide (PTIO), 100 µM 2,6-diaminonaftalene (DAN), and 1 mM l-arginine. When using antagonist for competition assays, cells are exposed to MAS1 antagonist A779 (D-Ala^7^-AT 1-7) at 0.1 µM for 15 min before addition of the cyclotides to be tested. Different concentrations of the cyclotides to be analyzed are added to the cell medium and the plates are agitated for 1 min before being placed into the incubator for 2 h. After 2 h, the cellular supernatants are transferred to opaque 96 well plates, and the amount of released NO is measured by fluorescence (λ_ex_ = 380 nm, λ_em_ = 425 nm).

### 4.8. Serum Stability

Human serum was spun down at 15,000 rpm for 10 min to separate the lipid components. Peptides (≈15 µg dissolved in 15 µL PBS (phosphate buffer saline)) were mixed with 150 µL human serum and incubated in a 37 °C water bath. Aliquot samples (15 µL) were taken at different time points and serum protein were precipitated with ≈70% MeCN at 4 °C for 10 min. The supernatant was separated, lyophilized, dissolved in 5% MeCN in water containing 0.1% formic acid, and analyzed by HPLC-MS/MS.

## 5. Conclusions

In conclusion, our results demonstrate for the first time the design of an engineered cyclotide using isopeptide peptide bonds able to activate the MAS1 receptor with low nanomolar activity and very high serum stability, thereby providing a promising lead compound for the design of novel therapeutics for the treatment of cancer and myocardial infarction.

## Supplementary Materials

Supplementary materials can be accessed at: http://www.mdpi.com/1420-3049/21/2/152/s1.

## References

[1] Gallagher P.E., Cook K., Soto-Pantoja D., Menon J., Tallant E.A. (2011). Angiotensin peptides and lung cancer. Curr. Cancer Drug Targets.

[2] Menon J., Soto-Pantoja D.R., Callahan M.F., Cline J.M., Ferrario C.M., Tallant E.A., Gallagher P.E. (2007). Angiotensin-(1–7) inhibits growth of human lung adenocarcinoma xenografts in nude mice through a reduction in Cyclooxygenase-2. Cancer Res..

[3] Gallagher P.E., Tallant E.A. (2004). Inhibition of human lung cancer cell growth by Angiotensin-(1–7). Carcinogenesis.

[4] Tallant E.A., Clark M.A. (2003). Molecular mechanisms of inhibition of vascular growth by Angiotensin-(1–7). Hypertension.

[5] Tallant E.A., Diz D.I., Ferrario C.M. (1999). State-of-the-art lecture. Antiproliferative actions of Angiotensin-(1–7) in vascular smooth muscle. Hypertension.

[6] Craik D.J., Daly N.L., Bond T., Waine C. (1999). Plant cyclotides: A unique family of cyclic and knotted proteins that defines the cyclic cystine knot structural motif. J. Mol. Biol..

[7] Daly N.L., Rosengren K.J., Craik D.J. (2009). Discovery, structure and biological activities of cyclotides. Adv. Drug Deliv. Rev..

[8] Gould A., Ji Y., Aboye T.L., Camarero J.A. (2011). Cyclotides, a novel ultrastable polypeptide scaffold for drug discovery. Curr. Pharm. Des..

[9] Puttamadappa S.S., Jagadish K., Shekhtman A., Camarero J.A. (2010). Backbone dynamics of cyclotide Mcoti-I free and complexed with trypsin. Angew. Chem. Int. Ed. Engl..

[10] Puttamadappa S.S., Jagadish K., Shekhtman A., Camarero J.A. (2011). Erratum in: Backbone dynamics of cyclotide mcoti-i free and complexed with trypsin. Angew. Chem. Int. Ed. Engl..

[11] Austin J., Kimura R.H., Woo Y.H., Camarero J.A. (2010). *In vivo* biosynthesis of an ala-scan library based on the cyclic peptide Sfti-1. Amino Acids.

[12] Huang Y.H., Colgrave M.L., Clark R.J., Kotze A.C., Craik D.J. (2010). Lysine-scanning mutagenesis reveals an amendable face of the cyclotide kalata B1 for the optimization of nematocidal activity. J. Biol. Chem..

[13] Simonsen S.M., Sando L., Rosengren K.J., Wang C.K., Colgrave M.L., Daly N.L., Craik D.J. (2008). Alanine scanning mutagenesis of the prototypic cyclotide reveals a cluster of residues essential for bioactivity. J. Biol. Chem..

[14] Felizmenio-Quimio M.E., Daly N.L., Craik D.J. (2001). Circular proteins in plants: Solution structure of a novel macrocyclic trypsin inhibitor from momordica cochinchinensis. J. Biol. Chem..

[15] Garcia A.E., Camarero J.A. (2010). Biological activities of natural and engineered cyclotides, a novel molecular scaffold for peptide-based therapeutics. Curr. Mol. Pharmacol..

[16] Contreras J., Elnagar A.Y., Hamm-Alvarez S.F., Camarero J.A. (2011). Cellular uptake of cyclotide Mcoti-I follows multiple endocytic pathways. J. Control. Release.

[17] Cascales L., Henriques S.T., Kerr M.C., Huang Y.H., Sweet M.J., Daly N.L., Craik D.J. (2011). Identification and characterization of a new family of cell-penetrating peptides: Cyclic cell-penetrating peptides. J. Biol. Chem..

[18] Ji Y., Majumder S., Millard M., Borra R., Bi T., Elnagar A.Y., Neamati N., Shekhtman A., Camarero J.A. (2013). *In vivo* activation of the P53 tumor suppressor pathway by an engineered cyclotide. J. Am. Chem. Soc..

[19] Henriques S.T., Craik D.J. (2010). Cyclotides as templates in drug design. Drug Discov. Today.

[20] Aboye T.L., Ha H., Majumder S., Christ F., Debyser Z., Shekhtman A., Neamati N., Camarero J.A. (2012). Design of a novel cyclotide-based Cxcr4 antagonist with anti-human immunodeficiency virus (HIV)-1 activity. J. Med. Chem..

[21] Chan L.Y., Gunasekera S., Henriques S.T., Worth N.F., Le S.J., Clark R.J., Campbell J.H., Craik D.J., Daly N.L. (2011). Engineering pro-angiogenic peptides using stable, disulfide-rich cyclic scaffolds. Blood.

[22] Sommerhoff C.P., Avrutina O., Schmoldt H.U., Gabrijelcic-Geiger D., Diederichsen U., Kolmar H. (2010). Engineered cystine knot miniproteins as potent inhibitors of human mast cell tryptase beta. J. Mol. Biol..

[23] Wang Y., Qian C., Roks A.J., Westermann D., Schumacher S.M., Escher F., Schoemaker R.G., Reudelhuber T.L., van Gilst W.H., Schultheiss H.P. (2010). Circulating rather than cardiac Angiotensin-(1–7) stimulates cardioprotection after myocardial infarction. Circ. Heart Fail.

[24] Loot A.E., Roks A.J., Henning R.H., Tio R.A., Suurmeijer A.J., Boomsma F., van Gilst W.H. (2002). Angiotensin-(1–7) attenuates the development of heart failure after myocardial infarction in rats. Circulation.

[25] Iusuf D., Henning R.H., van Gilst W.H., Roks A.J. (2008). Angiotensin-(1–7): Pharmacological properties and pharmacotherapeutic perspectives. Eur. J. Pharm..

[26] Hernandez J.F., Gagnon J., Chiche L., Nguyen T.M., Andrieu J.P., Heitz A., Trinh Hong T., Pham T.T., le Nguyen D. (2000). Squash trypsin inhibitors from momordica cochinchinensis exhibit an atypical macrocyclic structure. Biochemistry.

[27] Jagadish K., Gould A., Borra R., Majumder S., Mushtaq Z., Shekhtman A., Camarero J.A. (2015). Recombinant expression and phenotypic screening of a bioactive cyclotide against alpha-synuclein-induced cytotoxicity in baker’s yeast. Angew. Chem. Int. Ed. Engl..

[28] Jagadish K., Borra R., Lacey V., Majumder S., Shekhtman A., Wang L., Camarero J.A. (2013). Expression of fluorescent cyclotides using protein trans-splicing for easy monitoring of cyclotide-protein interactions. Angew. Chem. Int. Ed. Engl..

[29] Borra R., Camarero J.A. (2013). Recombinant expression of backbone-cyclized polypeptides. Biopolymers.

[30] Austin J., Wang W., Puttamadappa S., Shekhtman A., Camarero J.A. (2009). Biosynthesis and biological screening of a genetically encoded library based on the cyclotide Mcoti-I. Chembiochem.

[31] Durik M., van Veghel R., Kuipers A., Rink R., Haas Jimoh Akanbi M., Moll G., Danser A.H., Roks A.J. (2012). The effect of the thioether-bridged, stabilized Angiotensin-(1–7) analogue cyclic ang-(1–7) on cardiac remodeling and endothelial function in rats with myocardial infarction. Int. J. Hypertens.

[32] Kluskens L.D., Nelemans S.A., Rink R., de Vries L., Meter-Arkema A., Wang Y., Walther T., Kuipers A., Moll G.N., Haas M. (2009). Angiotensin-(1–7) with thioether bridge: An angiotensin-converting enzyme-resistant, potent Angiotensin-(1–7) analog. J. Pharmacol. Exp. Ther..

[33] Camarero J.A., Mitchell A.R. (2005). Synthesis of proteins by native chemical ligation using fmoc-based chemistry. Protein Pept. Lett..

[34] Cavanagh J., Rance M. (1992). Suppression of cross relaxation effects in tocsy spectra via a modified disi-2 mixing sequence. J. Magn. Res..

[35] Wuthrich K. (1986). NMR of Proteins and Nucleic Acids.

