# Supplementary material for: Design of a MCoTI-Based Cyclotide with Angiotensin (1-7)-Like Activity
Source: Molecules. 2016 Jan 26;21(2):152. doi: 10.3390/molecules21020152 (PMC4795166; doi:10.3390/molecules21020152)
Supplement: Supplementary file 1 [file molecules-21-00152-s001.pdf]

Supplementary Materials: Design of a MCoTI-Based Cyclotide with Angiotensin (1-7)-Like Activity

Teshome Aboye, Christopher J. Meeks, Subhabrata Majumder, Alexander Shekhtman, Kathleen Rodgers and Julio A. Camarero

Table S1. Tabulation of chemical shifts of backbone amide protons (δ ^{1}H-Nα and δ ^{1}Hα) protons of MCo-AT1-7 and their respective differences from cyclotide MCoTI-I.

Residue *	δ ^{1}H-Nα (ppm)	δ ^{1}Hα (ppm)	Δ δ ^{1}H-Nα (ppm)	Δ δ ^{1}Hα (ppm)
G1	8.112	3.988	0.001	0.021
G2	8.008	4.002	-0.034	0.169
V3	8.73	4.013	0.346	0.138
C4	8.665	5.13	0.083	0.064
P5	N/A ***	-	N/A ***	-
K6	8.138	4.177	−0.004	0.046
I7	7.541	4.177	−0.085	−0.081
L8	8.412	4.411	−0.177	0
Q9	8.602	4.267	−0.187	−0.195
R10	8.601	4.689	0.075	0.491 **
C11	8.185	4.71	−0.12	−0.043
R12	9.289	4.303	0.018	−0.019
R13	7.987	4.645	0.028	0
D14	9.065	3.956	−0.071	−0.029
S15	8.088	4.15	0.03	−0.021
D16	7.642	4.436	0.006	−0.07
C17	7.815	4.807	−0.194	−0.116
P18	N/A	-	N/A	-
G19	8.414	3.685	0.022	0.063
A20	8.412	4.314	0.089	−0.071
C21	7.994	4.478	−0.184	0.036
I22	8.899	4.289	−0.042	−0.021
C23	9.275	4.828	0.237	0.005
R24	8.013	4.171	0.064	−0.018
G25	8.975	3.813	0.165	0.011
N26	7.685	4.693	−0.014	0.113
G27	8.261	3.869	−0.061	0.01
Y28	7.202	5.127	0.028	−0.017
C29	8.668	5.29	−0.033	0.015
S30	9.685	3.842	−0.043	0
S31	8.672	4.468	−0.02	0.095
G32	8.900	4.008	−0.141	−0.302

* Sequence numbers are based on MCoTI-I; ** A rather large chemical shift difference (~0.5 ppm) of R10 alpha proton between MCo-AT1-7 and MCoTI-I is possibly induced by the concomitant changes in C11-C23 disulfide bridge related to grafting; *** Not available.

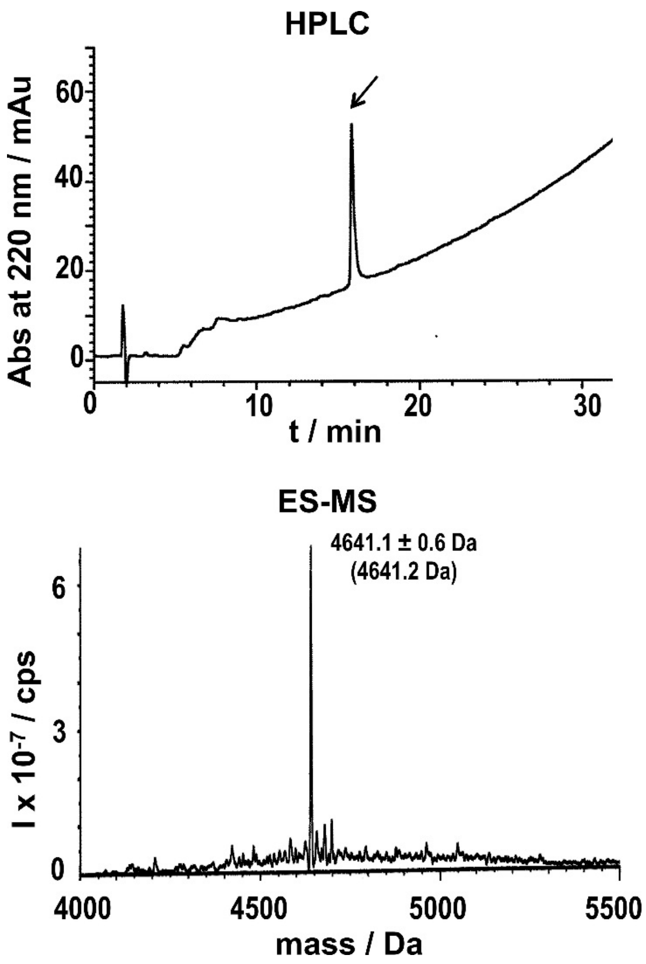

Figure S1. Analytical reverse-phase C18-HPLC trace and ESMS (deconvoluted) of linear S-alkylated MCo-AT1-7. HPLC analysis was performed using a linear gradient of 0%–70% solvent B over 30 min.

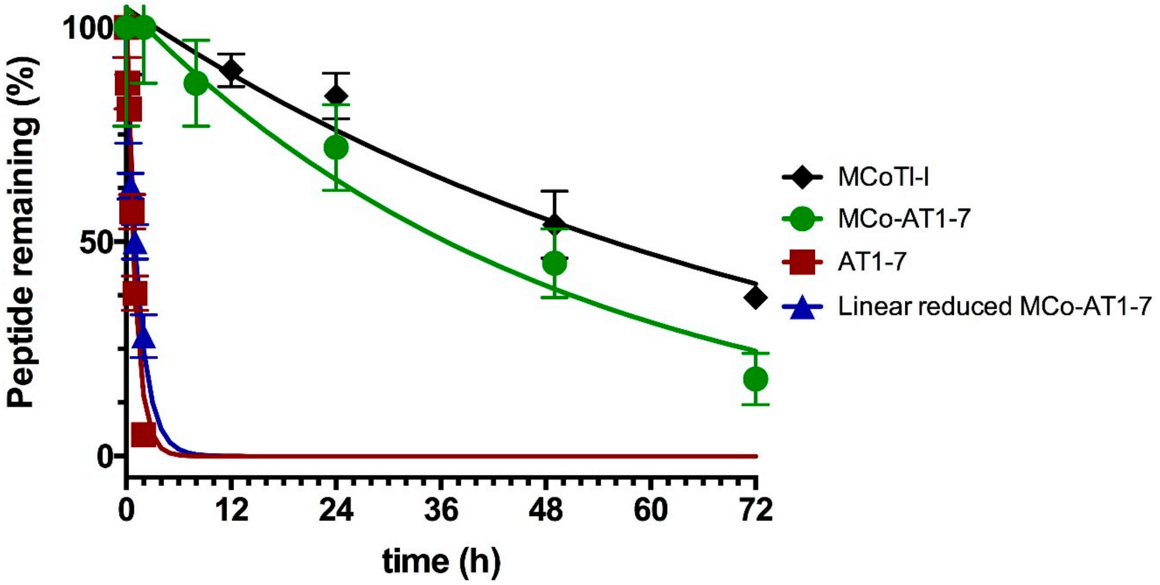

Figure S2. Stability of cyclotides MCo-AT1-7 and MCoTI-I; and peptides AT1-7 and reduced linear MCo-AT1-7 precursor to human serum at 37 °C. Undigested peptides were quantified by HPLC-MS/MS.

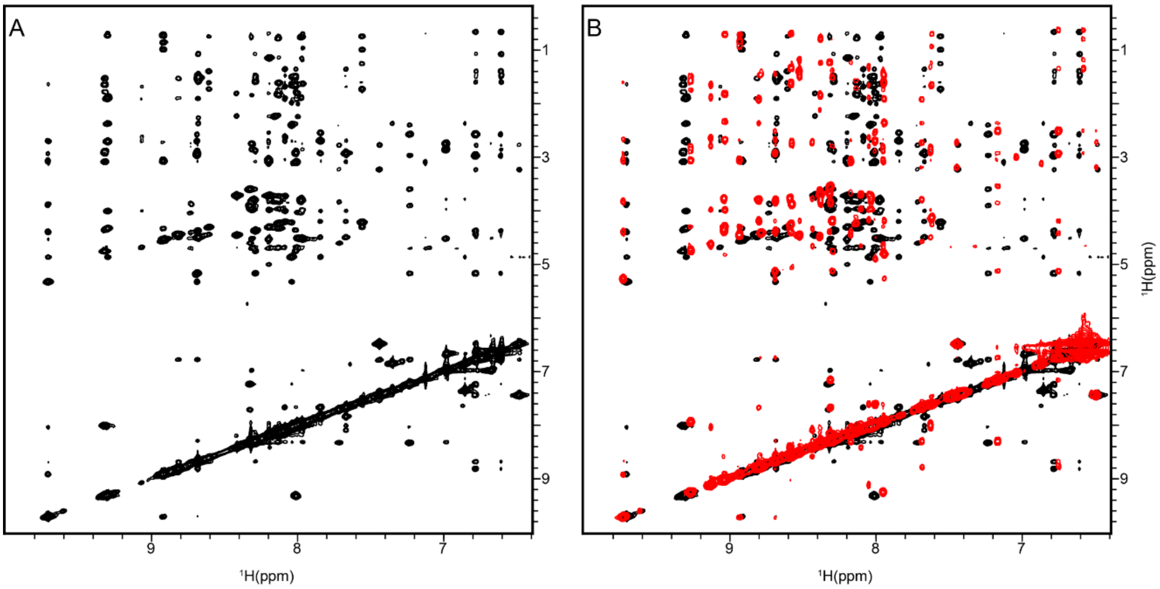

Figure S3. The MCo-AT1-7 fold is similar to that of MCoTI-I. (**A**) The amide protons from the $^1H\{^1H\}$-NOESY spectrum of MCo-AT1-7 are well dispersed (from 6.5 ppm to 9.8 ppm) and exhibit a large number of cross-peaks, which is indicative of a well-structured protein. (**B**) Overlay of the $^1H\{^1H\}$-NOESY spectra of MCo-AT1-7 (black) and MCoTI-I (red) shows that these two spectra are very similar; chemical shift differences of amide and/or alpha protons of the proteins are less than 0.2 ppm (Table S1).
